# “Corona-Debriefing”: concept and pilot testing of a 90-minute workshop for undergraduate-education and specialist-training in family medicine

**DOI:** 10.3205/zma001388

**Published:** 2020-12-03

**Authors:** Andreas Klement, Torben Ibs, Sebastian Longard, Catarina Klinkhart, Thomas Frese, Marcus Heise

**Affiliations:** 1 Universität Halle-Wittenberg, Institut für Allgemeinmedizin, KOMPAS: Kompetenzzentrum für die Weiterbildung in der Allgemeinmedizin Sachsen-Anhalt, Halle/Saale, Germany

**Keywords:** corona, debriefing, workshop, family medicine, pilottesting

## Abstract

**Background: **The corona pandemic is changing the framework conditions for medical studies and continuing education as well as the work with patients and within teams. Systematic reflection and communication about experiences and ways of dealing with them forms the basis for successful learning in and out of the crisis. Therefore, we designed a 90-minute workshop “Corona-Debriefing” for students and physicians in specialist-training in family medicine (ÄiW) using three successive moderated interaction phases: Questionnaire survey via tele-dialogue voting (TED) with immediate presentation of results and discussion, moderated experience reports on the categories risk/assessment/support/coping and finally moderated group discussions in small groups to collect “best practice” examples of crisis management.

**Objective: **We tested “Corona-Debriefing” as a pilot test with 48 participants (TN) in July 2020 (30 present, 14 online) in order to assess mental stress and risk perception of participants plus formative/brief summative evaluation of the workshop.

Methods: The PHQ-4 with its subscales GAD-2 (anxiety) and PHQ-2 (depression) was used to assess mental stress; risk assessments were made by means of self-constructed 5-point Likert-scales for the dimensions person/society/health/economy. A formative evaluation was carried out by means of a questionnaire at the end of the event; the brief summative assessment was asked for by means of a school grading scale.

**Results: **37 complete TED questionnaires and 22 evaluations were obtained. TN showed a low personal risk assessment, but considerable fears about social and economic developments. Needs are seen mainly in improvements regarding organization, protective equipment and technical communication (e.g. official recommendations for action). The workshop was rated “good” or “very good” in 95% of the evaluations. Criticism was directed at the limited time available, the narrowing of topics by moderators and the desire for (even) more room for the exchange of personal experiences.

**Conclusion: **The workshop “Corona-Debriefing” is a relatively easy way to use crisis experiences for learning processes. “Corona-Debriefing” can be used by changing the focus of moderation in various courses, years or fields of study, whereby the participants‘ own personal and clinical crisis experiences remain a prerequisite for a meaningful “debriefing”.

## Zusammenfassung

**Hintergrund: **Die Corona-Pandemie verändert die Rahmenbedingungen von Studium und Weiterbildung in der Medizin ebenso wie die Arbeit am Patienten und im Team. Eine systematische Reflektion und Kommunikation über Erfahrungen und Umgangsweisen bildet die Grundlage für ein erfolgreiches Lernen in und aus der Krise. Wir konzipierten daher einen 90-Minuten Workshop „Corona-Debriefing“ für Studierende und Ärzt*innen in Weiterbildung (ÄiW) mittels drei aufeinanderfolgenden moderierten Interaktionsphasen: Fragebogenerhebung per Tele-Dialog-Voting (TED) mit unmittelbarer Ergebnisvorstellung und Diskussion, moderierte Erlebnisberichte zu den Kategorien Gefährdung / Bewertung / Unterstützung / Bewältigung und schließlich moderierte Gruppendiskussionen in Kleingruppen zur Sammlung von „Best-Practice“ – Beispielen zur Krisenbewältigung.

**Ziele: **Wir erprobten „Corona-Debriefing“ als Pilottest mit 48 ÄiW als Teilnehmern (TN) im Juli 2020 (30 präsent, 14 online) mit den Auswertungszielen psychische Belastung / Gefährdungseinschätzung der TN, inhaltliche Schwerpunkte, technische Umsetzung, Gesamtbewertung und Kritik am Format.

Methoden: Zur Erfassung der psychischen Belastung diente der PHQ-4 mit seinen Subskalen GAD-2 (Ängstlichkeit) und PHQ-2 (Depressivität); Gefährdungseinschätzungen wurden mit selbstkonstruierten 5-Punkt-Likert-Skalen zu den Dimensionen Person/Gesellschaft/Gesundheit/Ökonomie erhoben. Eine formative und Evaluation erfolgte mittels Fragebogen am Veranstaltungsende, die Gesamtbewertung wurde mittels Schulnotenskala erfragt.

**Ergebnisse: **Ausgewertet werden konnten 37 komplette TED-Fragebögen und 22 Evaluationen. TN zeigten eine geringe persönliche Gefährdungseinschätzung, jedoch erhebliche Befürchtungen zu gesellschaftlichen und ökonomischen Entwicklungen. Bedarfe werden v.a. in Organisation, Schutzausrüstung und fachlicher Kommunikation (z.B. offiziellen Handlungsempfehlungen) gesehen. Der Workshop wurde in 95% der Evaluationen „gut“ oder „sehr gut“ bewertet. Kritik betraf zeitliche Enge, thematische Einengung durch Moderatoren und den Wunsch nach (noch) mehr Raum für Austausch persönlicher Erfahrungen.

**Schlussfolgerung: **Der Workshop „Corona-Debriefing“ ist eine relativ aufwandsarm umsetzbare Möglichkeit um Krisenerfahrungen für Lernprozesse zu nutzen. „Corona-Debriefing“ kann durch Änderung der Moderationsschwerpunkte in diversen Studiengängen, -jahren bzw. Fachgebieten genutzt werden, wobei eigene persönliche und klinische Krisenerfahrungen der TN Voraussetzung für ein sinnvolles „Debriefing“ bleiben.

## Introduction

The corona pandemic affects students and doctors in training both personally and professionally. Work and behavior in studies and at work had to adapt quickly to changing conditions [1]. Working conditions in family practice such as low-threshold-access, continuity and personal closeness were particularly affected [2]. Fears, insecurity and stress demanded special skills in self-care, communication and organization [3]. For successful learning in and out of crises, the concept of debriefing on the question “what has happened and how could we do better” is widely used. In medical didactics, systematic debriefing has so far been used mainly in simulation learning and dealing with critical events (e.g. CIRS), but it also appears to be suitable for dealing with crises as “facilitator-guided post-event debriefing” (PED) [4]. At our site, we have many years of experience with PED in the context of SkillsLab stations in medical studies. We now used PED to design a 90-minute workshop on “Lessons from Corona” in medical studies and specialist training in family practice, which asks for crisis experiences and strategies and collects “best practice”. We report on the pilot test among 48 residents in family medicine as poarticipants (TN) in July 2020. Due to corona-related distance rules, only 30 participants participated in presence (answers via WebTED) and another 18 online via BigBlueButton (answers via SoSciSurvey). PHQ-4 with its subscales GAD-2 (anxiety) and PHQ-2 (depressiveness) [5], which is commonly used in professionalization research on corona pandemics, served as a screening instrument for recording mental stress.

Evaluation objectives of the pilot test were mental stress/risk assessment of the participants as well as formative evaluation with focus on content, technical implementation and criticism of the format plus a brief summative evaluation.

## Project description

Usual components of a PED are an at least three-phase discussion structure for the description of reaction, analysis and processing of the event, mostly supplemented by a visualized summary of the learned [4]. We adapted the PED structure to a simultaneous presence and online format using multimedia tools (see table 1 (Tab. 1)).

## Results

Of 48 partcipants (TN) 70% were female; 75% ≤35 years old; 40% in their ≤2^nd^ and/or to 95% in ≤4^th^ year of residency. 37 questionnaires and 22 evaluations were obtained. SARS-CoV-2 contact was reported by 66% of the participants (of which 75% in professional context). Conspicuousness in PHQ-4 (≥3 points) showed three participants in the subscales for anxiety (GAD-2) and one participant in depression (PHQ-2). The mean values (standard deviations) for the sum indices of PHQ-4, PHQ-2 and GAD-2 were 2.00 (1.57); 0.78 (0.87) and 1.22 (0.90). Substantial effects of the PHQ dimensions were found in subjective experiences of psychosocial crises and risk assessment (especially social: health, economic & psychosocial) as well as in the need for professional information. The most strongly (“mostly” or “very”) affirmed questionnaire-items were social fears (-psychosocial 81%; economic 69%). 74% of participants felt that their choice of career was confirmed, but expressed clear needs for (better) organization (67.7%), equipment (58%) and information (44%). “Corona Debriefing" was rated as "good” or “very good” by 95% (n=21), only one participant rated for "not recommended". Criticism concerned temporal/thematic narrowness, more space for the exchange of personal experiences was (still) desired.

“Prompt narration” contributions came from about 20 of the 30 presence participants and five of 18 online participants (relatively 66 presence vs. 30% online contributions). The main narrative stimuli were support and stressful experiences. Initially, participants expressed increased uncertainty, tension and fears about the course of the crisis due to a lack of material (protective clothing), organizational (childcare) and informative (recommendations) support, culminating in anger and disappointment. However, this later on led into closer teamwork, development of (practical) individual solutions and a stronger sense of community, and even encouragement and pride. In the summary it could be stated: after initially percieved helplessness a solution-oriented reorganization in (practice) everyday life was possible, which led to more action security. An ambivalence between uncertainty on “what may come” and percieved personal confirmation remained. 

## Discussion

“Corona-Debriefing” was evaluated positively in the pilot test and – after establishing the technical prerequisites – could be implemented with little effort. In spite of many SARS-CoV-2 contacts, the TN showed population-like PHQ-4 values, which could be interpreted as an indicator of resilience. A large number of narratives on crisis experiences and strategies were stimulated. From this, “best practice” for mutual support and courses of action for the next wave/crisis could be abstracted – as usual in simulations [6] or recommended in reviews [7]. Individual personal and clinical crisis experiences of the participants form the basis for meaningful debriefing. Therefore, these must be explored in advance and the didactic concept and dimensions of the visualization must be adapted. The transfer of the concept to students, whose crisis experiences may differ from those of the TN, accordingly requires, in the sense of a “common experiential framework” [4], a change in the moderation focus (e.g. on learning methods; practical contacts) for impromptu narratives and small group work, as well as adaptation of some questionnaire items. Weaknesses regarding the significance of our pilot test are a lack of representativeness, moderator dependency and possibly differences in available technology as well as previous experiences of the participants. 

## Conclusion

“Corona-Debriefing” is a stimulating possibility for “learning in and out of the crisis”. A pilot test among residents in family medicine was very promising. The concept will be adapted for students in Halle-Wittenberg and re-evaluated in the comming term.

## Competing interests

The authors declare that they have no competing interests. 

## Figures and Tables

**Table 1:**
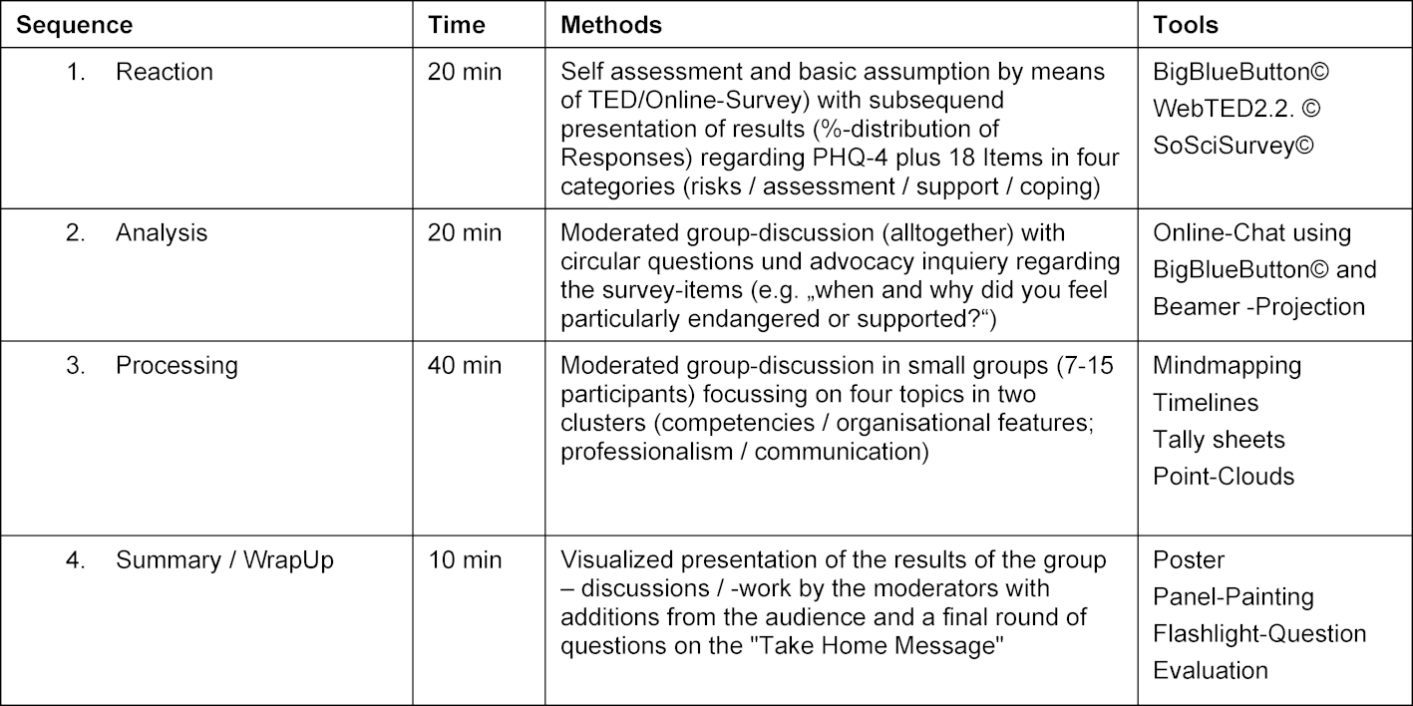
Corona-Debriefing: Concept for a 90-minute Workshop
